# Purine Analog-Like Properties of Bendamustine Underlie Rapid Activation of DNA Damage Response and Synergistic Effects with Pyrimidine Analogues in Lymphoid Malignancies

**DOI:** 10.1371/journal.pone.0090675

**Published:** 2014-03-13

**Authors:** Nobuya Hiraoka, Jiro Kikuchi, Takahiro Yamauchi, Daisuke Koyama, Taeko Wada, Mitsuyo Uesawa, Miyuki Akutsu, Shigehisa Mori, Yuichi Nakamura, Takanori Ueda, Yasuhiko Kano, Yusuke Furukawa

**Affiliations:** 1 Division of Stem Cell Regulation, Center for Molecular Medicine, Jichi Medical University, Shimotsuke, Tochigi, Japan; 2 Division of Hematology and Oncology, Faculty of Medical Sciences, University of Fukui, Eiheiji, Fukui, Japan; 3 Department of Hematology, Tochigi Cancer Center, Utsunomiya, Tochigi, Japan; 4 Medical Education Center, Saitama Medical University, Moroyama, Saitama, Japan; 5 Department of Hematology, Saitama Medical University, Moroyama, Saitama, Japan; Kanazawa University, Japan

## Abstract

Bendamustine has shown considerable clinical activity against indolent lymphoid malignancies as a single agent or in combination with rituximab, but combination with additional anti-cancer drugs may be required for refractory and/or relapsed cases as well as other intractable tumors. In this study, we attempted to determine suitable anti-cancer drugs to be combined with bendamustine for the treatment of mantle cell lymphoma, diffuse large B-cell lymphoma, aggressive lymphomas and multiple myeloma, all of which are relatively resistant to this drug, and investigated the mechanisms underlying synergism. Isobologram analysis revealed that bendamustine had synergistic effects with alkylating agents (4-hydroperoxy-cyclophosphamide, chlorambucil and melphalan) and pyrimidine analogues (cytosine arabinoside, gemcitabine and decitabine) in HBL-2, B104, Namalwa and U266 cell lines, which represent the above entities respectively. In cell cycle analysis, bendamustine induced late S-phase arrest, which was enhanced by 4-hydroperoxy-cyclophosphamide, and potentiated early S-phase arrest by cytosine arabinoside (Ara-C), followed by a robust increase in the size of sub-G1 fractions. Bendamustine was able to elicit DNA damage response and subsequent apoptosis faster and with shorter exposure than other alkylating agents due to rapid intracellular incorporation via equilibrative nucleoside transporters (ENTs). Furthermore, bendamustine increased the expression of ENT1 at both mRNA and protein levels and enhanced the uptake of Ara-C and subsequent increase in Ara-C triphosphate (Ara-CTP) in HBL-2 cells to an extent comparable with the purine analog fludarabine. These purine analog-like properties of bendamustine may underlie favorable combinations with other alkylators and pyrimidine analogues. Our findings may provide a theoretical basis for the development of more effective bendamustine-based combination therapies.

## Introduction

Bendamustine, 4-{5-[bis(2-chloroethyl)amino]-1-methyl-2-benzimidazolyl} butyric acid hydrochloride, is a bifunctional alkylating agent synthesized in the 60 s with the aim of combining the alkylating properties of 2-chloroethylamine and the antimetabolite properties of a benzimidazole ring [1]. Bendamustine is believed to act primarily as an alkylating agent that induces interstrand DNA cross-linking and subsequent strand breaks [2], but partial cross-resistance suggests a different mode of action between bendamustine and other alkylating agents such as cyclophosphamide, melphalan and cisplatin [3], [4]. Previous studies indicated the activation of DNA damage response and subsequent apoptosis, inhibition of mitotic checkpoints, and induction of mitotic catastrophe as the mechanisms of action of bendamustine [4]–[7]; however, most of them are shared with other alkylating agents and fail to explain the unique feature of this drug. It is likely that the purine analog-like structure contributes to the uniqueness of bendamustine, but this possibility has not yet been proven.

Bendamustine was used for the treatment of a variety of hematological and non-hematological malignancies between 1971 and 1992 in the German Democratic Republic [1]. Recent clinical trials in Europe and the United States confirmed the efficacy and safety of bendamustine as a single agent for chronic lymphocytic leukemia (CLL) [8] and rituximab-resistant low-grade lymphomas [9], and in combination with rituximab for patients with follicular lymphoma and mantle cell lymphoma [10], [11]. The spectrum of the clinical application of bendamustine is further expanding to diffuse large B-cell lymphoma (DLBCL) [12], aggressive lymphomas [13], [14], multiple myeloma [15], [16], T-cell lymphomas [17] and solid tumors [18], [19]. Although bendamustine monotherapy and the combination with rituximab appear to be successful for CLL and untreated indolent lymphomas [8], [11], combined chemotherapy with other therapeutic agents is required for the treatment of relapsed cases and refractory malignancies such as multiple myeloma and aggressive lymphomas.

Combined chemotherapy remains the primary approach for patients with hematological malignancies. The anti-cancer agents used for combination are generally selected on the basis of single-agent activity, non-overlapping toxicity, and the lack of cross-resistance and antagonistic interaction. In addition, mechanistic insight is important for the establishment of effective and safe regimens. In the case of bendamustine, its unique mechanisms of action may influence the selection of drugs to be combined. Previous preclinical studies have demonstrated the combined effects of bendamustine with cytosine arabinoside, gemcitabine, fludarabine, cladribine, mitoxantrone, doxorubicin and entinostat [5], [6], [20]–[24]. Some of the combinations have been clinically translated with anticipated success [25]–[28], but theoretical basis of their effects requires independent validation. To establish more effective and safer regimens, we systematically screened for suitable drugs to be combined with bendamustine for intractable lymphoid malignancies and investigated the mechanisms underlying favorable combinations in the present study. Among lymphoid malignancies, we focused on mantle cell lymphoma, DLBCL, Burkitt lymphoma and multiple myeloma, because of their relative resistance to bendamustine monotherapy in clinical settings [12]–[16]. We found that bendamustine made favorable combinations with alkylating agents and pyrimidine analogues in these tumors at least partly due to its purine analog-like properties. This finding may provide important information for the establishment of effective bendamustine-based regimens.

## Materials and Methods

### Drugs

Bendamustine was provided by SymBio Pharmaceuticals Ltd. (Tokyo, Japan). Other anti-cancer agents used and their sources are 4-hydroperoxy-cyclophosphamide (4-OHCY; an active metabolite of cyclophosphamide) (Shionogi, Osaka, Japan), chlorambucil (LKT Laboratories, St. Paul, MN, USA), melphalan (Wako Biochemicals, Osaka, Japan), cytosine arabinoside (Ara-C) (Nihon Shinyaku, Kyoto, Japan), gemcitabine (Eli Lilly, Kobe, Japan), decitabine (Sigma-Aldrich, St. Louis, MO, USA), 9-ß-D-arabinosyl-2-fluoroadenine (F-Ara-A; an active metabolite of fludarabine) (Sigma-Aldrich), doxorubicin (Meiji, Tokyo, Japan), mitoxantrone (Lederle Japan, Tokyo, Japan), etoposide (Nihon Kayaku, Tokyo, Japan), methotrexate (Lederle Japan), vincristine (Shionogi) and bortezomib (LC Laboratories, Wobum, MA, USA). Dilazep (N,N’-bis-(E)-[5-(3,4,5-trimethoxy-baenzoate)-4-pentenyl] homopiperazine) was provided by Kowa Pharmaceuticals (Tokyo, Japan). S-(4-nitrobenzyl)-6-thioinsine (NBTI) was purchased from Sigma-Aldrich.

### Cell Lines

We used two multiple myeloma (U266 and RPMI 8226), two Burkitt lymphoma (BJAB and Namalwa), four mantle cell lymphoma (HBL-2, SMCH-16, Granta519 and NCEB-1), two diffuse large B-cell lymphoma (TK and B104), two T-cell acute lymphoblastic leukemia (Jurkat and KOPT-5) and three acute myeloid leukemia (HL-60, K562 and THP-1) cell lines for drug sensitivity screening. These were purchased from the Health Science Research Resources Bank (Osaka, Japan) except for mantle cell lymphoma cell lines [29], [30].

### Cell Proliferation Assay

Cells were harvested at the logarithmic phase and resuspended at 1–5×10^5^ cells/ml in RPMI1640 medium containing 10% fetal bovine serum. After overnight culture in a humidified atmosphere of 95% air/5% CO2 at 37°C, drug solutions were added and cells were further incubated for given culture periods. Viable cell numbers were estimated by the reduction of 3-(4,5-dimethylthiazol-2-yl)-2,5-diphenyltetrazolium bromide (MTT) using a Cell Counting Kit (Wako Biochemicals). Absorbance at 450-nm (A450) was determined with a microplate reader and expressed as a ratio of the value of corresponding untreated cells.

### Drug Combination Study

To analyze cytotoxic interactions, we cultured cells in the presence of 0, 20, 40, 60, 80 and 100% of IC50 and IC80 doses of bendamustine and another drug simultaneously for 96 hours. The combined effects were evaluated by the isobologram method of Steel and Peckham as described previously [31], [32]. In brief, three isoeffect curves are constructed based on the dose-response curve of bendamustine and another drug. If two agents act additively by independent mechanisms, their combined data points will lie near the line of hetero-addition. If agents act additively by similar mechanisms, their combined data points will lie near the lines of iso-addition (Figure S1). Because the difference in IC levels did not affect the conclusions, we present only the results of the IC80 level. We statistically analyzed overall effects of drug combination using Wilcoxon signed-rank test. If the observed values are significantly (*P*<0.05) smaller than the predicted minimum values, the combination is regarded as synergistic. If *P* values are greater than 0.05, the combination is regarded as additive/synergistic. If the observed data fall between the predicted minimum and maximum values, the combination is regarded as additive.

### Cell Cycle Analysis

The cell cycle profile was obtained by staining DNA with Vindelov’s solution (0.04 mg/ml propidium iodide in 5 mM Tris-HCl, 5 mM NaCl and 0.005% Nonidet P-40) in preparation for flow cytometry with the FACScan/CellFIT system (Becton-Dickinson, San Jose, CA). The size of the sub-G1, G0/G1 and S+G2/M fractions was calculated as a percentage by analyzing DNA histograms with the ModFitLT 2.0 program (Becton-Dickinson).

### Cell Culture

We examined the effect of ENT1 inhibitors on anti-cancer drugs according to previous reports [33]. In brief, HBL-2 and Namalwa cells were cultured in the absence or presence of IC50 doses of cytosine arabinoside, F-Ara-A, bendamustine and 4-OHCY (10, 2.5, 25 and 2 µM, respectively) with various concentrations of either dilazep or NBTI for 72 hours. Relative cytotoxic effects were calculated according to the following formula: 1- (A450 in the presence of both drugs and inhibitors/A450 in the presence of inhibitors alone)/1- (A450 in the presence of drugs alone/A450 in the presence of inhibitors alone) × 100.

We compared the combined effects of bendamustine and cytosine arabinoside between simultaneous and sequential additions. In the former, HBL-2 cells were cultured in the presence of various concentrations of the two drugs for 48 hours. In case of sequential additions, HBL-2 cells were cultured with various concentrations of either cytosine arabinoside or bendamustine for 48 hours, washed with phosphate-buffered saline, resuspended in the complete medium containing various concentrations of either bendamustine or cytosine arabinoside, and cultured for additional 48 hours. Isobolograms with then generated from dose-response curves obtained under each condition.

### Immunoblotting

HBL-2 and Namalwa cells were cultured in the absence or presence of IC50 doses of each drug. Whole cell lysates were isolated at given time points and subjected to immunoblot analysis using specific antibodies against phosphorylated Chk1 at Ser-296, phosphorylated Chk2 at Thr-68 (Cell Signaling Technology, Beverly, MA), ENT1 (F-12), ENT2 (H-46) and GAPDH (FL-335) (Santa Cruz Biotechnology, Santa Cruz, CA) [34].

### Real-time Quantitative RT-PCR

HBL-2 and Namalwa cells were cultured in the absence or presence of IC50 doses of 4-OHCY, bendamustine or F-Ara-A (2, 25 and 2.5 µM, respectively). Total cellular RNA was isolated after 48 hours using the RNeasy Kit (QIAGEN, Valencia, CA) and reverse-transcribed into cDNA using ReverTra Ace and oligo (dT) primers (TOYOBO, Tokyo, Japan). We performed real-time quantitative RT-PCR using the TaqMan Gene Expression Assay System (Hs01085704 for *SLC29A1/ENT1* and Hs01922876 for *GAPDH*) with TaqMan Fast Universal PCR Master Mix (Applied Biosystems, Warrington, UK) as described previously [35]. The data were quantified with the 2^−ΔΔCt^ method using simultaneously amplified *GAPDH* as a reference.

### Measurement of Ara-C and F-Ara-A Uptake

We measured cellular uptake of Ara-C and F-Ara-A using [5-^3^H]Ara-C and [8-^3^H]F-Ara-A (Moravek Biochemicals, Brea, CA, USA) as described previously [36]. Briefly, HBL-2 cells (1×10^6^ cells/ml) were incubated with 10 µM F-Ara-A or bendamustine for 3 h at 37°C, followed by washing into fresh media and subsequent incubation with either [5-^3^H]Ara-C or [8-^3^H]F-Ara-A at 10 µM (30 Ci/mmol) for 6 h at 37°C. The samples were then centrifuged to collect the cell pellets (400×*g*, 10 min, 4°C). The acid-soluble fraction, the nucleotide pool, was extracted by adding perchloric acid, followed by neutralization with KOH, and subjected to scintillation counting for radioactivity detection.

### Determination of Intracellular Ara-CTP

HBL-2 cells (1×10^6^ cells/ml, 10 ml) were incubated with or without 10 µM (final concentration) F-Ara-A or 10 µM (final concentration) bendamustine for 3 h at 37°C, followed by washing into fresh media and subsequent incubation with 10 µM (final concentration) Ara-C for 6 h at 37°C. The acid-soluble fraction was prepared as described above. The intracellular active metabolite of Ara-C, Ara-CTP, was determined as described previously [37]. Briefly, the samples were subjected to isocratic high-performance liquid chromatography (HPLC) using a TSK gel DEAE-2 SW column (length, 250 mm; internal diameter, 4.6 mm) (Tosoh, Tokyo, Japan) and 0.06 M Na2HPO4 (pH 6.9) −20% acetonitrile buffer (a constant flow rate of 0.7 ml/min and at ambient temperature). The Ara-CTP peak was identified by its retention time and quantitated from its peak area at an absorbance of 269 nm.

## Results

### Bendamustine Induces Apoptosis Faster than other Alkylating Agents but does not Exert Sufficient Cytotoxicity against all Tumors

**Figure 1:**
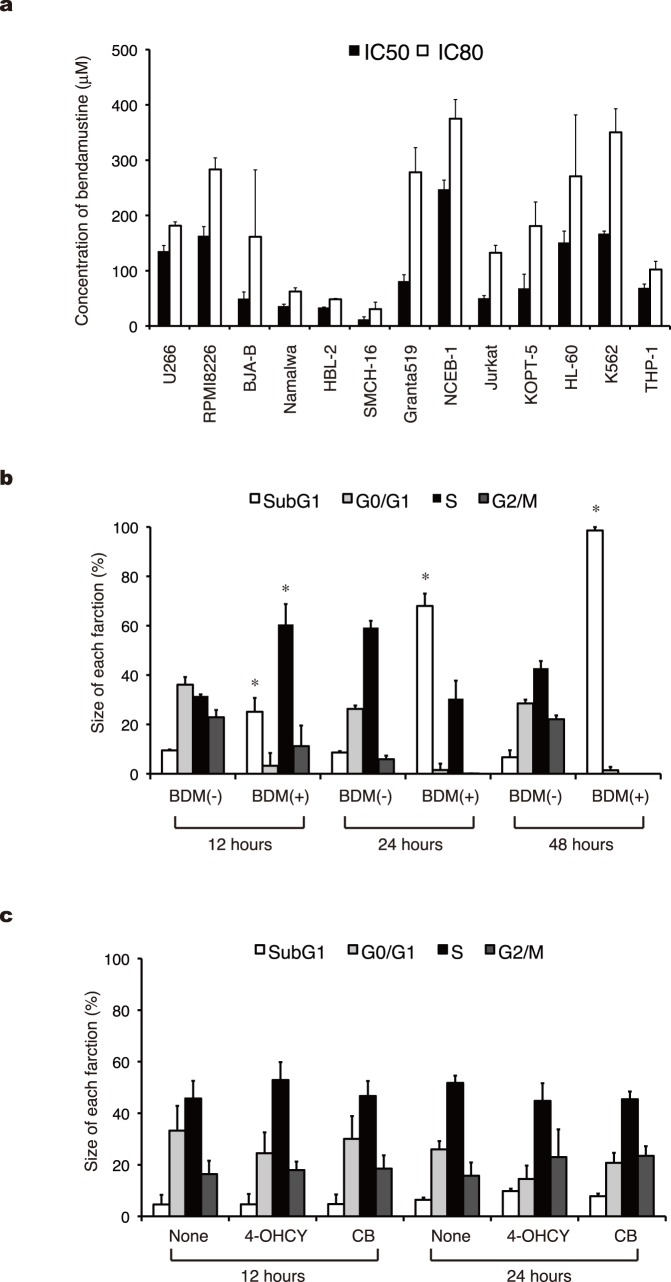
Bendamustine induces apoptosis faster than other alkylating agents but does not exert sufficient cytotoxicity against all tumors. A) We cultured the indicated cell lines with various concentrations of bendamustine and measured cell proliferation with the MTT reduction assay after 72 hours. IC50 and IC80 values are defined as the concentrations of drugs that produce 50 and 80% inhibition of cell growth, respectively. The means ± S.D. (bars) of three independent experiments are shown. B) HBL-2 cells were cultured in the absence (−) or presence (+) of the IC50 value of bendamustine (BDM), harvested at the indicated time points, and stained with propidium iodide in preparation for cell cycle analysis. C) HBL-2 cells were cultured in the absence (None) or presence of IC50 values of 4-OHCY or chlorambucil (CB), harvested at the indicated time points, and stained with propidium iodide in preparation for cell cycle analysis. Columns indicate the quantification of cells in each phase of the cell cycle obtained with the ModFitLT 2.0 program. The means ± S.D. (bars) of three independent experiments are shown. *P*-values were calculated by one-way ANOVA with the Student-Newman-Keuls multiple comparisons test. Asterisks denote *p*<0.05 against the untreated control.

Bendamustine has a unique anti-tumor spectrum according to the *In Vitro* Cell Line Screening Project (IVCLSP) and National Cancer Institute (NCI) COMPARE analyses [4]. In this study, we first attempted to confirm the unique pattern of cytotoxicity in hematologic malignancies. As shown in Figure 1A, bendamustine displayed considerable cytotoxicity against cell lines derived from mantle cell lymphoma (HBL-2 and SMCH16), Burkitt lymphoma (BJAB and Namalwa) and T-cell acute lymphoblastic leukemia (Jurkat and KOPT-5), whereas the effects on acute myeloid leukemia and myeloma cell lines were relatively weak. In addition, the DLBCL cell lines, TK and B104, had intermediate sensitivity to bendamustine with IC50 values of 47.0±4.6 and 42.0±6.9 µM, respectively. It is of note that two of four mantle cell lymphoma cell lines (Granta519 and NCEB-1) were highly resistant to this drug.

To understand the nature of bendamustine-mediated growth inhibition, we analyzed the cell cycle pattern of bendamustine-treated HBL-2 and Namalwa cells. The IC50 value of bendamustine (25 µM) induced S-phase arrest at an early time point (12 hours), followed by a time-dependent increase in the size of sub-G1 fractions (Figure 1B). On the other hand, the IC50 values of 4-OHCY and chlorambucil neither induced cell cycle arrest nor increased the size of sub-G1 fractions within 24 hours (Figure 1C). As the sub-G1 fraction is caused by apoptosis-specific DNA fragmentation, these results indicate that bendamustine induces S-phase arrest and subsequent apoptosis faster than other alkylating agents. The induction of apoptosis was independently confirmed by annexin-V staining and caspase-3 activation (data not shown).

### The Selection of Suitable Drugs to be Combined with Bendamustine for Intractable Lymphoid Malignancies using Isobologram

Drug sensitivity screening revealed that the IC50 values of sensitive and resistant cell lines were 10–30 µM and 100–250 µM, respectively. This clearly indicates that combination with other anti-cancer agents is essential for the treatment of bendamustine-insensitive tumors, because bendamustine yielded a maximum serum concentration of approximately 25 µM after intravenous administration of the usual dose (120 mg/m^2^) with a mean elimination half-life of 30–50 minutes [38], [39]. We therefore analyzed cytotoxic interactions between bendamustine and 13 drugs that represent six different classes of cytotoxic agents in lymphoid malignancies relatively resistant to bendamustine monotherapy in clinical settings: mantle cell lymphoma (HBL-2), diffuse large B-cell lymphoma (B104), Burkitt lymphoma (Namalwa) and multiple myeloma (U266). To quantify cytotoxic interactions, we constructed isobolograms with three isoeffect curves (mode I and mode II lines) from dose-response curves of bendamustine and the combined drugs using data points at the IC80 and IC50 levels (Figure S1).

**Figure 2:**
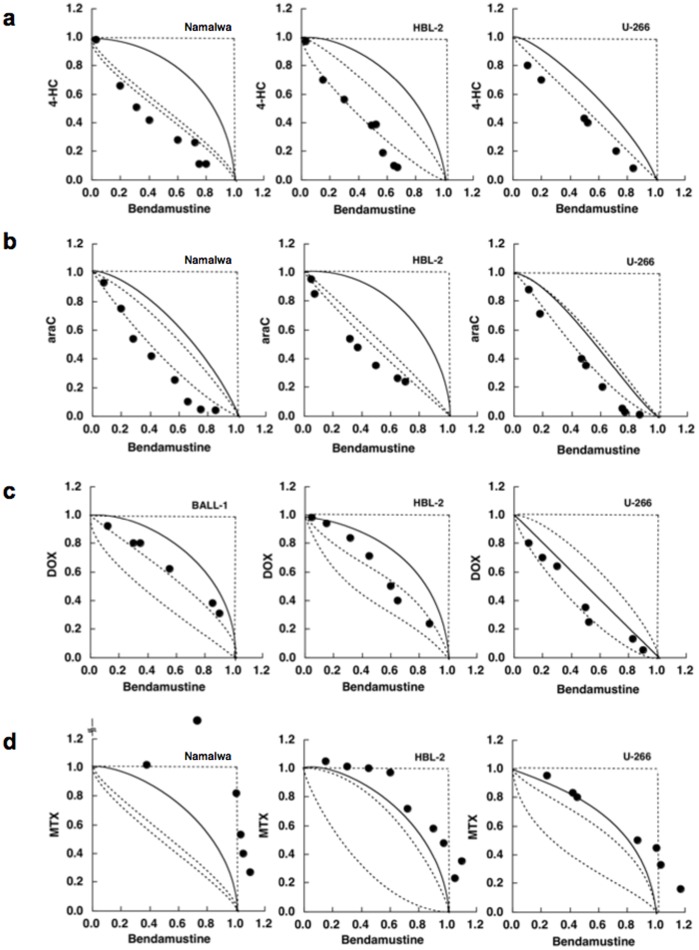
The selection of suitable drugs to be combined with bendamustine using isobologram. Cells were cultured with various concentrations of bendamustine in combination with (A) 4-hydroperoxy-cyclophosphamide (4-HC), (B) cytosine arabinoside (araC), (C) doxorubicin (DOX) and (D) methotrexate (MTX) for 4 days (Namalwa and HBL-2) or 7 days (U266). Isobolograms were generated from dose-response curves of each combination as described previously [31], [32]. The results of data quantification and statistical analysis are shown in Table 1.

Figure 2A shows the representative isobolograms of the combination of bendamustine and 4-OHCY, in which all or most data points for the combination fell in the area of supra-additivity in all cell lines tested. The mean values of observed data were significantly smaller than those of the predicted minimum values for the additive effect in B104, Namalwa and U266, indicating a synergistic effect of the two drugs (Table 1). Similar results were obtained in combination with bendamustine and other alkylating agents such as chlorambucil and melphalan (data not shown). Figure 2B shows the isobolograms of the combination of bendamustine and cytosine arabinoside, in which all or most data points fell in the area of supra-additivity in all cell lines tested. The mean values of the observed data were significantly smaller than those of the predicted minimum values for the additive effect, indicating a synergistic effect of the two drugs (Table 1). The combination of bendamustine and two other pyrimidine analogues, gemcitabine and decitabine, produced virtually identical results, whereas the combination with a purine analogue F-Ara-A was only additive (Table 1). The combination of bendamustine and topoisomerase inhibitors (doxorubicin, mitoxantrone and etoposide) yielded additive effects in all cell lines examined (Figure 2C and Table 1). It is of note that bendamustine and bortezomib made favorable combinations (Table 1). In contrast, methotrexate was quite antagonistic with bendamustine (Figure 2D and Table 1). These results suggest that alkylating agents and pyrimidine analogues are suitable drugs to be combined with bendamustine for the treatment of intractable lymphoid malignancies.

**Table 1:** Quantitative analysis of the combination of bendamustine and other drugs in lymphoid malignancies.

Combined drugs	Cell lines	Data points	Observed data*	Predicted mini.**	Predicted max.***	Effects#
4-OHCY	HBL-2	8	0.44	0.47	0.81	additive/synergistic
	B104	4	0.47	0.58	0.82	synergistic
	Namalwa	5	0.38	0.51	0.79	synergistic
	U266	6	0,55	0.62	0.75	synergistic
Ara-C	HBL-2	7	0.45	0.49	0.83	synergistic
	B104	5	0.44	0.55	0.79	synergistic
	Namalwa	5	0.51	0.63	0.80	synergistic
	U266	8	0.68	0.74	0.86	synergistic
Gemcitabine	HBL-2	7	0.37	0.45	0.92	synergistic
	B104	4	0.40	0.51	0.93	synergistic
	Namalwa	6	0.39	0.45	0.78	synergistic
	U266	7	0.35	0.45	0.82	synergistic
Decitabine	HBL-2	5	0.47	0.61	0.89	synergistic
	B104	4	0.41	0.52	0.74	synergistic
	Namalwa	7	0.45	0.48	0.84	additive/synergistic
	U266	7	0.39	0.57	0.78	synergistic
F-Ara-A	HBL-2	8	0.42	0.36	0.90	additive
	B104	4	0.48	0.41	0.83	additive
	Namalwa	4	0.59	0.55	0.77	additive
	U266	7	0.53	0.33	0.85	additive
Doxorubicin	HBL-2	6	0.63	0.42	0.86	additive
	B104	4	0.59	0.48	0.81	additive
	Namalwa	5	0.68	0.35	0.78	additive
	U266	7	0.62	0.54	0.84	additive
Mitoxantrone	HBL-2	7	0.55	0.52	0.86	additive
	B104	4	0.55	0.43	0.66	additive
	Namalwa	5	0.61	0.38	0.72	additive
	U266	7	0.52	0.51	0.57	additive
Etoposide	HBL-2	9	0.61	0.42	0.87	additive
	B104	4	0.59	0.48	0.84	additive
	Namalwa	4	0.65	0.57	0.80	additive
	U266	9	0.65	0.53	0.95	additive
Methotrexate	HBL-2	9	0.93	0.22	0.80	antagonistic
	B104	4	0.98	0.44	0.75	antagonistic
	Namalwa	5	1.02	0.45	0.68	antagonistic
	U266	7	0.71	0.36	0.68	antagonistic
Vincristine	HBL-2	7	0.60	0.40	0.74	additive
	B104	4	0.55	0.42	0.71	additive
	Namalwa	5	0.59	0.46	0.77	additive
	U266	6	0.57	0.40	0.72	additive
Bortezomib	HBL-2	11	0.44	0.58	0.81	additive/synergistic
	B104	4	0.53	0.59	0.88	additive/synergistic
	Namalwa	5	0.62	0.53	0.79	synergistic
	U266	6	0.87	0.63	0.99	additive

*Mean values of observed data (S.D. not shown).

**Mean values of the predicted minimum values for an additive effect (S.D. not shown).

***Mean values of the predicted maximum values for an additive effect (S.D. not shown).

# Overall effect of drug combination (see Materials and Methods for the method of evaluation).

### Cell Cycle Effects of the Combination of Bendamustine with Cyclophosphamide or Cytosine Arabinoside

**Figure 3:**
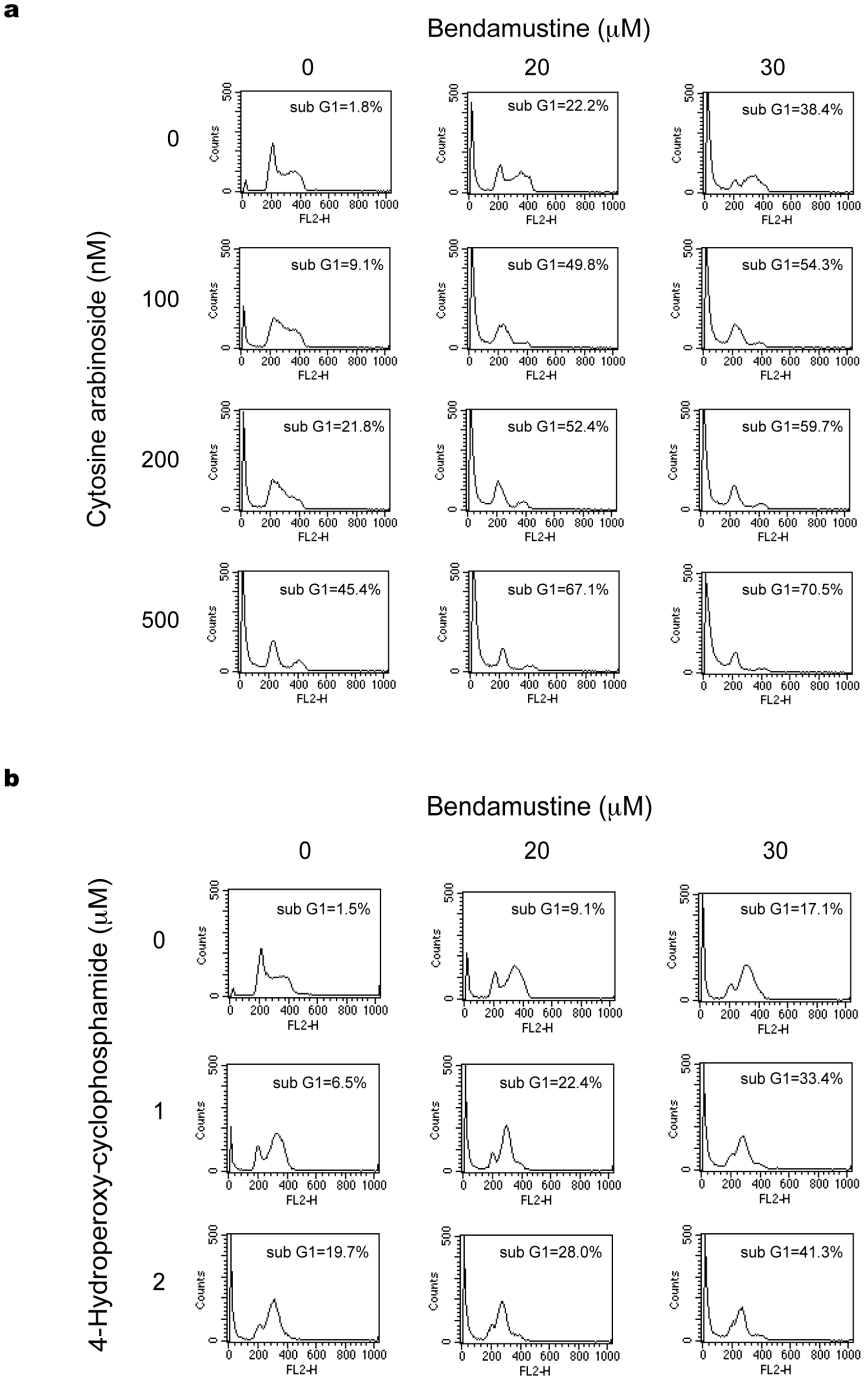
Cell cycle effects of the combination of bendamustine with 4-OHCY or cytosine arabinoside. (A) HBL-2 cells were cultured with bendamustine alone, cytosine arabinoside alone or their combination for 48 hours. (B) HBL-2 cells were cultured with bendamustine alone, 4-OHCY alone or their combination for 48 hours. Cell cycle profiles were obtained by flow cytometry as described in Materials and Methods. The size of the sub-G1 fraction was calculated by analyzing DNA histograms with the ModFitLT 2.0 program. The data shown are representative of multiple independent experiments with various concentrations of the drugs.

Next, we attempted to clarify the mechanisms by which alkylating agents and pyrimidine analogues are synergistic with bendamustine. Toward this end, we first performed cell cycle analysis of HBL-2 cells treated with bendamustine in combination with either 4-OHCY or cytosine arabinoside. Bendamustine alone arrested target cells in the late S phase, whereas cytosine arabinoside caused early S-phase block in HBL-2 cells (Figure 3A). The combination of the two drugs induced a decrease in late S-phase cells with massive apoptosis. As shown in Figure 3B, 4-OHCY alone arrested cells in mid- to late S phase 48 hours after culture. Simultaneous addition of bendamustine and 4-OHCY enhanced S-phase arrest, followed by an increase in the size of sub-G1 fractions. The results of cell cycle analysis imply that bendamustine and 4-OHCY exert synergistic effects by activating the same pathway, probably DNA damage response, leading to enhanced S-phase arrest and apoptosis, whereas bendamustine and cytosine arabinoside might potentiate each other in different ways to yield synergism.

**Figure 4:**
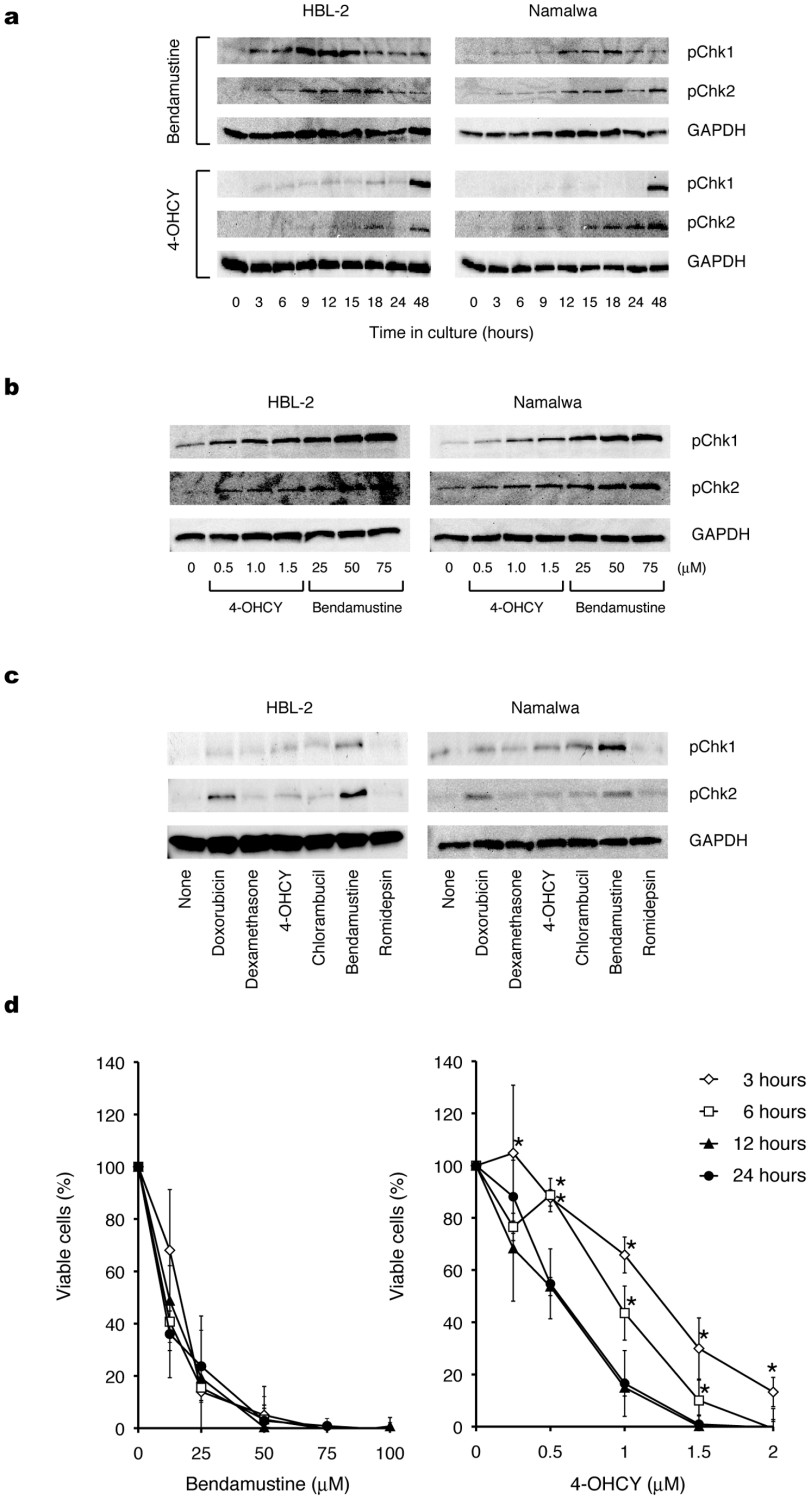
Bendamustine elicits DNA damage response and subsequent apoptosis faster and with a shorter exposure time than other alkylating agents. (A) Time-course analysis of Chk1 and Chk2 phosphorylation in HBL-2 and Namalwa cells treated with IC50 values of bendamustine or 4-OHCY. (B) Dose-response analysis of Chk1 and Chk2 phosphorylation in HBL-2 and Namalwa cells treated with bendamustine or 4-OHCY for 12 hours. (C) Chk1 and Chk2 phosphorylation was detected in HBL-2 and Namalwa cells treated with IC50 values of the indicated drugs for 6 hours. The membranes were reprobed with anti-GAPDH antibody to serve as a loading control in each experiment. The data shown are representative of multiple independent experiments. (D) After treatment for the indicated periods (3–24 hours) with the indicated doses of bendamustine or 4-OHCY, HBL-2 cells were washed twice with fresh medium and cultured in complete medium without drugs. The cells were cultured for 72 hours in total and subjected to MTT assays. Panels show the dose-response curves of bendamustine- and 4-OHCY-treated cells. The means ± S.D. (bars) of three independent experiments are shown. *P*-values were calculated by one-way ANOVA with the Student-Newman-Keuls multiple comparisons test. Asterisks indicate *p*<0.05 against each value of 24 h exposure.

### Bendamustine Elicits DNA Damage Response and Subsequent Apoptosis Faster and with a Shorter Exposure Time than other Alkylating Agents

If bendamustine and 4-OHCY could exert synergistic effects by enhancing the same pathway, this might be linked to the ability of bendamustine to induce DNA damage (S-phase arrest) and apoptosis rapidly, as shown in Figure 1B. To confirm this hypothesis, we investigated whether bendamustine indeed activates DNA damage response faster than other alkylating agents. For this purpose, we compared the kinetics of checkpoint kinase activation by bendamustine with that of 4-OHCY. As shown in Figure 4A, bendamustine induced marked phosphorylation of checkpoint kinases Chk1 and Chk2 in HBL-2 and Namalwa cells at early time points (3–18 hours), whereas the equitoxic dose of 4-OHCY failed to do so at the same time points. In bendamustine-treated cells, Chk1 and Chk2 phosphorylation peaked at 9–18 hours, whereas it peaked after 48 hours with 4-OHCY treatment at equitoxic concentrations. To confirm the above finding, we cultured HBL-2 and Namalwa cells with various concentrations of bendamustine and 4-OHCY for 12 hours and found that bendamustine induced stronger phosphorylation than 4-OHCY in an equitoxic range (Figure 4B). In support of these observations, bendamustine induced the phosphorylation of ATM and p53 markedly and ATR slightly in HBL-2 cells after 6 and 3 hours, respectively, whereas 4-OHCY induced very weak or negligible phosphorylation of DNA damage response proteins under the same condition (Figure S2). Furthermore, we examined the phosphorylation of Chk1 and Chk2 in HBL-2 and Namalwa cells treated with IC50 values of various anti-cancer agents for 6 hours. As shown in Figure 4C, bendamustine readily induced the phosphorylation of Chk1 and Chk2, whereas other drugs could not provoke comparable levels of phosphorylation at this time point.

These results indicate that bendamustine can rapidly induce irreparable DNA damage, thereby triggering Chk1- and Chk2-dependent apoptosis faster than other alkylating agents. To corroborate this assumption, we performed wash-out experiments and found that only 3-hour exposure was sufficient for bendamustine to elicit full cytotoxic activity in HBL-2 cells (Figure 4D, left panel), whereas 4-OHCY required at least 12-hour exposure (Figure 4D, right panel). These observations suggest that the exposure time required for commitment to cell death is very short for bendamustine, explaining the additive effects of bendamustine and other alkylating agents; DNA damage rapidly provoked by the former (within 24 hours) is boosted later by the latter (after 48 hours). However, additional evidence is required to explain the synergism between bendamustine and other alkylators. Nonetheless, an emerging question here is why bendamustine can induce DNA damage more rapidly than other alkylating agents.

### Purine Analog-like Properties Underlie Rapid Induction of DNA Damage and Synergistic Effects with Pyrimidine Analogues

**Figure 5:**
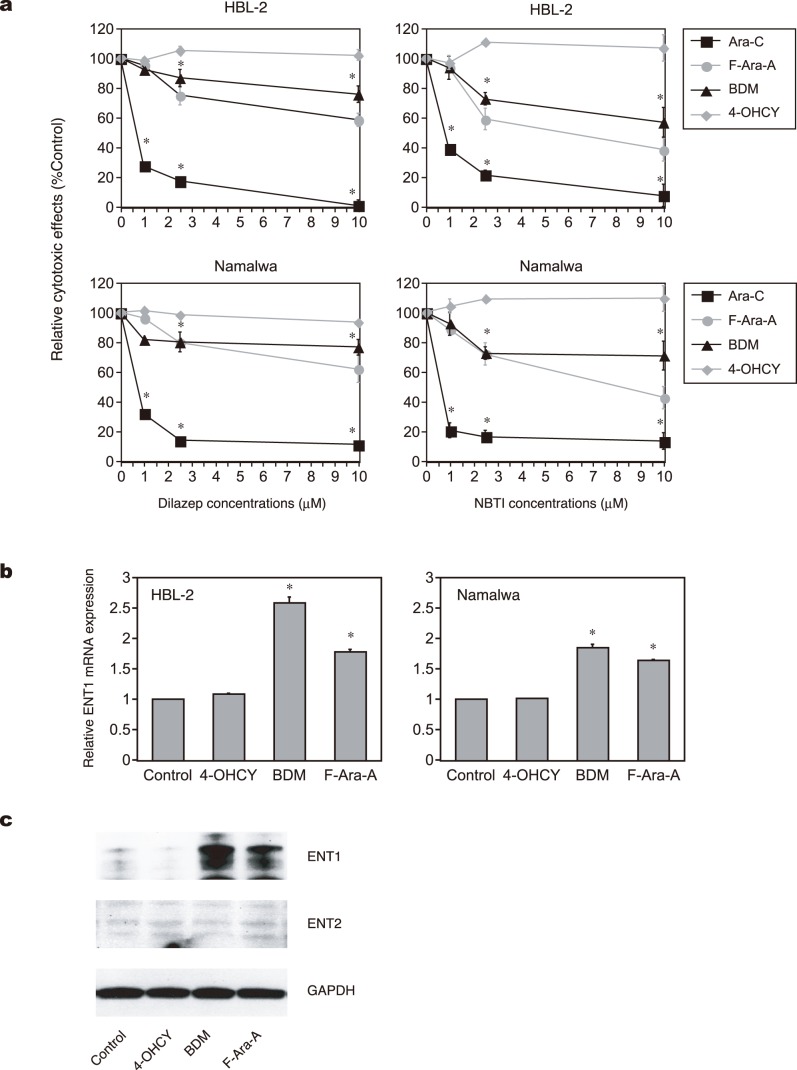
Purine analog-like properties of bendamustine. (A) Effects of dilazep (left panel) and NBTI (right panel) on cytotoxicity of the indicated drugs at IC50 against HBL-2 (upper panel) and Namalwa (lower panel) cells. (B) *ENT1* mRNA expression in HBL-2 and Namalwa cells treated with the indicated drugs. The y-axes indicate relative gene expression against the expression levels of the untreated control being set at 1.0. The means ± S.D. (bars) of three independent experiments are shown. *P*-values were calculated by one-way ANOVA with the Student-Newman-Keuls multiple comparisons test. Asterisks denote *p*<0.05 against the untreated control. (C) HBL-2 and Namalwa cells were cultured in the absence (Control) or presence of IC50 values of the indicated drugs. Whole cell lysates were isolated after 48 hours and subjected to immunoblot analysis for the expression of ENT1, ENT2 and GAPDH (internal control). The data shown are representative of multiple independent experiments.

Rapid uptake of the drug may provide a good explanation for the rapid induction of DNA damage by bendamustine. In general, uptake of alkylating agents is mediated through simple passive diffusion [40], [41]. In addition to simple passive diffusion, bendamustine uptake might be facilitated via nucleoside transporters because of its purine-like structure [42], [43]. This possibility was proposed in a preliminary study [44], but has not been confirmed to date. We tested this possibility using dilazep, a potent inhibitor of both equilibrative nucleoside transporter 1 (ENT1) and ENT2, and NBTI, a specific inhibitor of ENT1 (33, 42, 43). As anticipated, both dilazep and NBTI almost completely abrogated the cytotoxic effect of cytosine arabinoside against HBL-2 and Namalwa cells, whereas they did not affect the activity of 4-OHCY at all (Figure 5A). Under the same experimental condition, the effect of bendamustine was slightly but significantly ameliorated by both inhibitors to a similar extent as that of a *bona fide* purine analog F-Ara-A. These results suggest that cellular uptake of bendamustine is at least partly mediated through nucleoside transporters, which enable rapid internalization and activation of DNA damage response.

**Figure 6:**
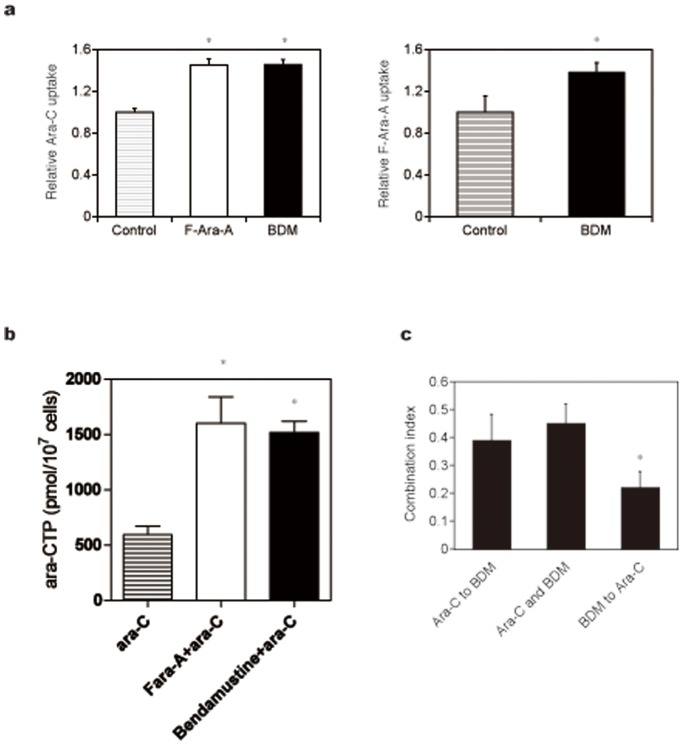
Bendamustine enhances the uptake of Ara-C and subsequent increase in Ara-CTP in HBL-2 cells. (A) HBL-2 cells were pretreated with the vehicle alone (Control), F-Ara-A or bendamustine (BDM), followed by the incubation with either [5-^3^H]Ara-C (left panel) and [8-^3^H]F-Ara-A (right panel). Drug incorporation was estimated by counting radioactivity of the nucleotide pool. (B) HBL-2 cells were pretreated with the vehicle alone (ara-C), F-Ara-A (F-ara-A+ara-C) or bendamustine (Bendamustine+ara-C), followed by the incubation with Ara-C. Intracellular Ara-CTP levels were determined using HPLC as described in Materials and Methods. (C) HBL-2 cells were treated with Ara-C and bendamustine (BDM) under three different conditions as described in Materials and Methods and subjected to isobologram analysis to compare the combination index. The means ± S.D. (bars) of three independent experiments are shown. *P*-values were calculated by one-way ANOVA with the Student-Newman-Keuls multiple comparisons test. Asterisks denote *p*<0.05 against the untreated control.

It is well known that purine analogs potentiate the activity of cytosine arabinoside by increasing intracellular concentrations of the drug and its active metabolite Ara-CTP [45], [46]. In addition, Petersen et al. [47] reported that purine analogs auto-enhanced the cytotoxic effects by up-regulating the expression of nucleoside transporters in CLL cells. From these observations, we reasoned that bendamustine exerts synergistic effects with pyrimidine analogues via modulation of ENT expression. As shown in Figure 5B and 5C, bendamustine readily increased the expression of ENT1 but not ENT2 at both mRNA and protein levels to an extent comparable with F-Ara-A. In accord with the increased expression of ENT1, cellular uptake of its substrates, cytosine arabinoside and F-Ara-A, was significantly enhanced by pretreatment with bendamustine (Figure 6A). Furthermore, bendamustine actually increased the intracellular concentration of Ara-CTP, an active metabolite of cytosine arabinoside, in HBL-2 cells (Figure 6B). If bendamustine potentiates the activity of cytosine arabinoside by enhancing the expression of ENT1, pretreatment with bendamustine produces more potent effects than simultaneous addition of both agents. The results shown in Figure 6C indicate that this is really the case; sequential addition of bendamustine followed by cytosine arabinoside yielded significantly stronger synergism than simultaneous addition of both agents and sequential addition of cytosine arabinoside followed by bendamustine.

## Discussion

The efficacy of bendamustine monotherapy and its combination with rituximab has been established in the treatment of CLL and untreated indolent lymphomas [8], [11]; however, combined therapy with other therapeutic agents might be required for the treatment of relapsed cases and intractable malignancies such as mantle cell lymphoma, DLBCL, aggressive lymphomas and multiple myeloma, all of which are relatively resistant to bendamustine. In this study, we therefore investigated the interactions between bendamustine and 13 drugs that represent six different classes of cytotoxic agents commonly used for the treatment of lymphoid malignancies in cell lines derived from bendamustine-resistant entities. We found that bendamustine yielded particularly effective combinations with alkylating agents (4-hydroperoxy-cyclophosphamide, chlorambucil and melphalan) and pyrimidine analogues (cytosine arabinoside, gemcitabine and decitabine), and determined that purine analog-like properties of bendamustine underlie the synergic interactions.

As it is widely believed that bendamustine primarily functions as an alkylating agent, the synergistic effect with other alkylators seems to be unreasonable. We propose different kinetics of the DNA damage response as a mechanism of favorable combination. Bendamustine is rapidly incorporated into target cells through nucleoside transporters, probably because of its purine-like structure, thereby inducing DNA damage significantly faster than others. DNA damage rapidly provoked by bendamustine could be boosted later by other alkylating agents. Moreover, biological half-lives of bendamustine and cyclophosphamide are 49.1 and 311.4 minutes, respectively [38], [39], [48]. Therefore, rapid transport of bendamustine is advantageous for active forms to be accumulated in target cells more efficiently, resulting in rapid and robust induction of DNA damage, followed by the effects of other agents with longer half-lives such as cyclophosphamide. Although this scenario may explain additive effects, further investigation is required to understand the mechanism of the synergism between bendamustine and other alkylating agents.

The purine analog-like properties of bendamustine also provide a good explanation for its synergistic effects with pyrimidine analogues. Purine analogs are known to potentiate the activity of cytosine arabinoside by increasing intracellular concentrations of the drug and its active metabolite Ara-CTP via inhibition of ribonucleotide reductase [45], [46] and enhancement of ENT expression [47]. We found that bendamustine also induced the up-regulation of ENT1 expression and an increase in Ara-CTP in target cells, which underlies the synergistic effects with bendamustine and cytosine arabinoside. Simultaneous addition of bendamustine and F-Ara-A, another substrate of ENT1, yielded only an additive effect in isobologram analysis. This may be due to the competition of the two agents for ENT1, because pretreatment with bendamustine significantly enhanced the accumulation of F-Ara-A, which administered later, in HBL-2 cells. It is of note that bendamustine-induced increase in ENT1 expression occurs at mRNA levels. This is compatible with the results of a previous Gene Ontology study, in which bendamustine could up-regulate the expression of multiple and distinct sets of genes, including those related to nucleobase, nucleoside, nucleotide and nucleic acid metabolism, compared with other alkylating agents [4]. The mechanisms underlying the up-regulation of ENT1 transcripts by bendamustine are currently under investigation in our laboratory.

Some clinical trials have documented the efficacy of the combination of bendamustine and other drugs, such as mitoxantrone, fludarabine, cytosine arabinoside, vincristine and corticosteroids, for patients with relapsed and/or refractory lymphoid malignancies [25]–[28], [49]. Among them, the combination of bendamustine with cytosine arabinoside (R-BAC therapy) showed a remarkable therapeutic impact with moderate toxicity on patients with CLL and mantle cell lymphoma ineligible for intensive treatments [27], [28]. The synergistic effect of bendamustine and cytosine arabinoside is fully consistent with our observation and others [22], [23]. Furthermore, in the R-BAC regimen, sequential treatment with bendamustine first followed by cytosine arabinoside was proven to be more effective than simultaneous addition of the two drugs. This clinical fact is well supported by our experimental findings. In addition, the combination of bendamustine with cytosine arabinoside and melphalan (BeEAM) is highly efficacious as a conditioning regimen to stem cell transplantation for heavily treated patients with Hodgkin lymphoma, DLBCL and mantle cell lymphoma [50]. Undoubtedly, such effective regimens are in high demand for intractable malignancies including mantle cell lymphoma and multiple myeloma. The present findings provide a theoretical basis for the development of more effective bendamustine-based combination therapies.

## Supporting Information

Figure S1 **Schematic representation of the isobologram of Steel and Peckham.** Envelope of additivity, surrounded by Mode I (solid line) and Mode II (dotted lines) isobologram lines, was constructed from the dose-response curves of bendamustine and a combined drug. The concentrations that produced 80% or 50% growth inhibition were expressed as 1.0 on the ordinate and the abscissa of isobolograms. Combined data points Pa, Pb, Pc and Pd represent supra-additive, additive, sub-additive and protective effects, respectively.
(TIF)

Figure S2 **Time-course analysis of ATM, ATR and p53 phosphorylation in HBL-2 cells treated with IC50 values of bendamustine or 4-OHCY.** We used specific antibodies against phosphorylated p53 at Ser-15, phosphorylated ATM at Ser-1981 and phosphorylated ATR at Ser-428 (Cell Signaling Technology). The membranes were reblotted with anti-GAPDH antibody to serve as an internal control.
(TIF)
