# Supplementary figures and images for: Purine Analog-Like Properties of Bendamustine Underlie Rapid Activation of DNA Damage Response and Synergistic Effects with Pyrimidine Analogues in Lymphoid Malignancies
Source: PLoS One. 2014 Mar 13;9(3):e90675. doi: 10.1371/journal.pone.0090675 (PMC3953125; doi:10.1371/journal.pone.0090675)

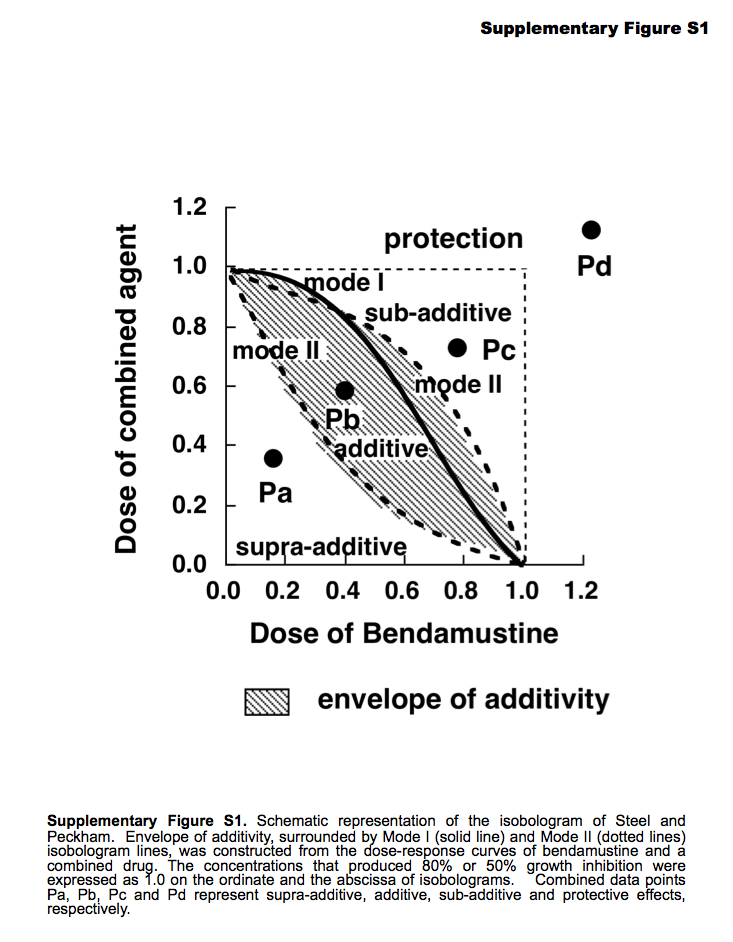

Supplement: Figure S1 — Schematic representation of the isobologram of Steel and Peckham. Envelope of additivity, surrounded by Mode I (solid line) and Mode II (dotted lines) isobologram lines, was constructed from the dose-response curves of bendamustine and a combined drug. The concentrations that produced 80% or 50% growth inhibition were expressed as 1.0 on the ordinate and the abscissa of isobolograms. Combined data points Pa, Pb, Pc and Pd represent supra-additive, additive, sub-additive and protective effects, respectively. (TIF) [file pone.0090675.s001.tif]

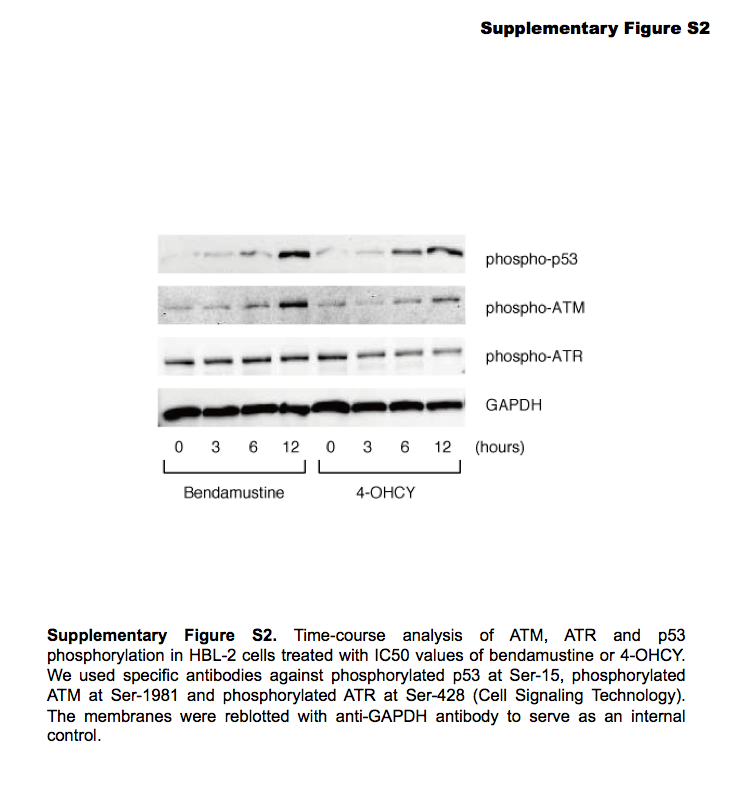

Supplement: Figure S2 — Time-course analysis of ATM, ATR and p53 phosphorylation in HBL-2 cells treated with IC50 values of bendamustine or 4-OHCY. We used specific antibodies against phosphorylated p53 at Ser-15, phosphorylated ATM at Ser-1981 and phosphorylated ATR at Ser-428 (Cell Signaling Technology). The membranes were reblotted with anti-GAPDH antibody to serve as an internal control. (TIF) [file pone.0090675.s002.tif]
